# An Eco‐Friendly Synthesis and Characterization of Antibacterial, Antifungal, and Antioxidant Silver Nanoparticles From Bioactive *Streptomyces* sp. Strain WSN‐2

**DOI:** 10.1002/mbo3.70209

**Published:** 2025-12-28

**Authors:** Muhammad Sultan Anjum, Shazia Khaliq, Neelma Ashraf, Munir Ahmad Anwar, Kalsoom Akhtar

**Affiliations:** ^1^ Industrial Biotechnology Division, National Institute for Biotechnology and Genetic Engineering (NIBGE) Constituent College of Pakistan Institute of Engineering and Applied Sciences (PIEAS) Jhang Road, PO Box 577 Faisalabad 38000 Pakistan; ^2^ Institute of Pharmaceutical Sciences, Pharmaceutical Biology and Biotechnology Albert‐Ludwig University of Freiburg Stefan‐Meier‐Str. 19 (VF) Freiburg im Breisgau 79104 Germany

**Keywords:** antibacterial, antifungal, nanomedicine, Silver nanoparticles, *Streptomyces* sp. strain WSN‐2

## Abstract

The present study reports the isolation and molecular identification of *Streptomyces* sp. strain WSN‐2 using 16S rRNA gene sequencing and BLASTn analysis (GenBank Accession No. MN128377), followed by its application in the green synthesis of silver nanoparticles (AgNPs). Biomass filtrate of *Streptomyces* sp. WSN‐2 efficiently reduced silver ions to form stable AgNPs, confirmed by a characteristic UV‐Vis surface plasmon resonance (SPR) peak at 423 nm. Structural and morphological characterization using FTIR, SEM, TEM, and EDX revealed spherical nanoparticles with a smooth texture and well‐dispersed arrangement. TEM analysis indicated particle size predominantly between 50 and 60 nm (overall range 0.83–100 nm), while the zeta potential of –22.9 mV confirmed moderate colloidal stability. EDX spectra displayed strong elemental silver absorption peaks at 3‐4 keV, indicating crystalline Ag formation. The biosynthesized AgNPs exhibited strong antimicrobial activity against wide range of pathogenic microbes. Maximum antibacterial growth inhibition zones were observed against *S. typhi* (24 ± 1.53 mm), followed by *E. coli* (23 ± 1.25 mm), *B. subtilis* (23 ± 1.73 mm), and *P. aeruginosa* (22 ± 1.53 mm). Antifungal assays revealed highest antifungal activity against *A. flavus* (16 ± 1.15 mm), and notable inhibition of *A. niger* (16 ± 1.25 mm), *A. fumigatus* (15 ± 1.70 mm), and *F. oxysporum* (14 ± 1.53 mm). MIC values ranged from 8.00 ± 0.05 µg/mL for *P. aeruginosa* to 18.000.07 µg/mL for *A. fumigatus*. The AgNPs also demonstrated remarkable antioxidant potential, achieving 65.2% H₂O₂ scavenging activity at 50 µg/mL, surpassing L‐ascorbic acid (45.1%). These findings highlight *Streptomyces* sp. WSN‐2 as a promising biogenic source for the synthesis of stable AgNPs with significant antibacterial, antifungal, and antioxidant potential.

## Graphical abstract

Green‐synthesized AgNPs from *Streptomyces* sp. WSN‐2 with robust antibacterial, antifungal, and antioxidant properties, revealing a powerful and eco‐friendly route for producing bioactive nanomaterials.

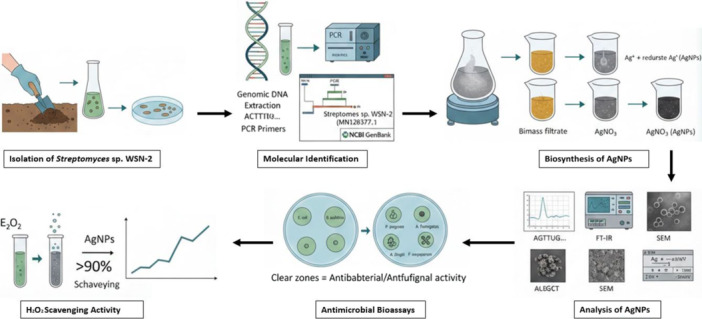

## 1 Introduction

Silver nanoparticles (AgNPs) have been recognized as an attractive agent in the field of biomedical and agriculture due to their broad‐spectrum antimicrobial characteristics, easy synthesis, and controllable physiochemical properties (Amar kumar et al. 2024; Li et al. 2022). Green synthesis of AgNPs via microorganisms has great benefits in contrast to chemical or physical methods because biological method is economical, utilize milder conditions, and produce biocompatible encapsulations obtained from biomolecules such as proteins, polysaccharides, enzymes, which ultimately preserves nanoparticles and their biological activity for longer times (Ahmad et al. 2024). Among the list of microbes, use for the green synthesis of nanoparticles, *Streptomyces* species belongs to class actinomycetes greatly known for their potential of producing antimicrobials as well as extracellular enzymes (Ashraf et al. 2022; Jain et al. 2021). *Streptomyces* spp. have now considered to be nano‐factories for AgNPs production due to their extracellular secondary metabolites for instance, reductases, and proteins (Prabhahar et al. 2022). These metabolites are able to reduce both Ag⁺ and cap the nanoparticles that ultimately generates stable, bioactive silver nanoparticle (AgNPs) (Nejad et al. 2024). Researchers reported the production strategies and biosynthetic processes about the *Streptomyces* based AgNPs that are commonly extracellular using culture filtrate or cell free supernatant and intracellular (using whole cells) (Murugesan et al. 2024). Whereas, extracellular strategy is considered easier for downstream purification. Moreover, the factors like AgNO₃ concentration, biomass/filtrate ratio, pH, temperature, and incubation time greatly influence the particle nucleation, formation and size or shape (Shahzadi et al. 2025). However, number of studies reported systematic optimization of these factors specifically for *Streptomyces* strains ranging from 10 to 80 nm for small spherical to aggregated AgNPs with instant color change and properties surface plasmon resonance (SPR) peaks in UV‐Vis spectra (ranging from 400 to 450 nm) (Anjum et al. 2024; Abd‐Elhady et al. 2021).

Previous studies reported number of techniques for the characterization of *Streptomyces* derived AgNPs such as, UV‐Vis spectroscopy for SPR, transmission electron microscopy (TEM) and scanning electron microscopy (SEM) for morphology and size, dynamic light scattering (DLS) for hydrodynamic size and polydispersity, X‐ray diffraction (XRD) for crystalline phase, energy‐dispersive X‐ray spectroscopy (EDX) for elemental composition (Dayma et al. 2024). Moreover, Fourier‐transform infrared (FTIR) spectroscopy used to characterize the bio‐molecular capping agents, and zeta‐potential to visualize colloidal stability. Many researchers reported that biomolecule capping from *Streptomyces* filtrates produces negative zeta potentials (enhanced stability) and FTIR signatures consistent with proteins/amide bonds proved that extracellular proteins/enzymes play their role both as reducing as well as capping agents (Gemishev et al. 2019; Aryani et al. 2025). The antimicrobial properties of *Streptomyces* derived AgNPs has been confirmed repeatedly against Gram‐positive and Gram‐negative bacteria (e.g., *Staphylococcus aureus*, *Escherichia coli*, *Klebsiella pneumoni*ae, *Pseudomonas aeruginosa*) and fungal pathogens (e.g., *Candida* spp. and various phyto‐pathogenic fungi) (Sethi et al. 2024; Ashraf et al. 2021). Although, the mechanism includes AgNPs attachment to the bacterial cells, disrupts the cell wall or membrane that ultimately increase the permeability, formation of the reactive oxygen species (ROS) and oxidative stress, or release of Ag⁺ ions that interferes with thiol groups present in proteins and DNA. Additionally, it interacts with respiratory enzymes and metabolic reactions (More et al. 2023). Various *Streptomyces* derived AgNPs reported, dose dependent inhibition zones, low minimum inhibitory concentrations (MICs) in comparison to bulk silver salts, antibiofilm activity, and synergistic effects when mixed with some antibiotics (AboElmaaty et al. 2022).

However, studies from recent years have depicted number of applications and boosted biological evaluation of *Streptomyces* derived AgNPs. Moreover, some studies (Paliwal and Paliwal 2025) reported the enhanced antifungal activity of *Streptomyces* derived AgNPs against the plant pathogens indicating the agro‐protectant potential of AgNPs, additionally, antibacterial and antibiofilm effects related to medical instrument coatings, in vitro toxicological assessment to visualize the therapeutic threshold and multi‐functional testing such as antioxidant, wound healing effects and anti‐cancerous potential for biomedical applications (Sambangi and Gopalakrishnan 2023). These studies clearly focus on the precise characterization and standardized antimicrobial assays for instance, MIC, MBC, time kill kinetics curves, biofilms inhibition as well as mode‐of‐action experiments (ROS assays, membrane integrity). Despite promising results, this discourse also puts light on the challenges that need to be address before translational deployment such as batch to batch variations because of the changes in the biological material, lack of knowledge related to accurate bio‐molecular reducers or capping agents, due to the high risk of cytotoxicity, and environmental hazards. Although, optimization of the synthesis protocol, scale up studies, vigorous toxicological assessment including both in vitro and *in vivo*, and mechanistic insights of *Streptomyces* metabolites that mediate the reduction and capping are active areas of research. Moreover, various reviews and comparative researches indicated the adoption of optimized culture conditions and post synthetic surface functionalization can control antimicrobial potency while, mitigating the non‐targeted toxicity (Herdiana et al. 2022; Pognan et al. 2023).

To conclude, *Streptomyces* spp. are considered a gold mine for the synthesis of AgNPs with antibacterial and antifungal potential. While, previous studies revealed the reproducibility of AgNPs, characterization methodology, consistent antimicrobial efficacy against the diversified pathogens that make AgNPs attention seeking agents to the safety perspectives and practical applications. Consequently, continuous research should pay focus on clarity of mechanism, scale up standardization to shift *Streptomyces* based AgNPs towards real world applications. Owing to the aforementioned perspectives in consideration, the present study was undertaken to synthesize and characterize AgNPs utilizing *Streptomyces* sp. strain WSN‐2. Furthermore, the study aimed to assess the potential of these AgNPs against a range of pathogenic bacterial and fungal strains.

## 2 Materials and Methods

### 2.1 Isolation and Screening of *Streptomyces* sp. Strain WSN‐2

The samples were collected from the rhizospheric soil from a depth of 5–10 cm from various locations in Faisalabad, Pakistan regions (31°25′05.10′N 73°04′39.27′E) and then brought back to the laboratory under sterile conditions, where they were stored at 4°C ± 2°C for further analysis. Subsequently, the samples were subjected to a 10‐fold serial dilution method to achieve a minimum colony density. The final dilutions were used for the isolation of *Streptomyces* sp. in sterilized International *Streptomyces* Project medium (ISP2) (0.5 g K_2_HPO_4_, 3 g Casein, 10 g Starch, 1 g Peptone, 1 g Yeast extract, 10 g Malt extract, 25 g Agar) with specific modifications (Sanjivkumar et al. 2019). After an incubation period of 7 days at 30°C, the isolated strains were utilized for subsequent experiments particularly, for the synthesis of nanoparticles. Among all the strains, one potent strain coded as WSN‐2 was selected for further investigations.

### 2.2 Molecular Identification of *Streptomyces* sp. Strain WSN‐2

The extraction of genomic DNA of strain WSN‐2 was conducted using a previously optimized protocol with specific modifications (Lee et al. 2003). Subsequently, the purity and quantity of the extracted DNA were assessed using a Thermo Scientific 2000 spectrophotometer. The region of the 16S rRNA gene was amplified through PCR reaction mixture (50 µL), which included 85 ng of template DNA and universal primers, namely FD1 (5′ AGT TTG ATC CTG GCT CAG 3′) and RP1 (5′ ACG GCT ACC TTG TTA CGA CTT 3′).

The purified PCR product was sequenced by Macrogen (South Korea), and the sequence obtained was compared with homologous sequences using NCBI BLAST. The sequences showing more than 95% identities were aligned with query sequence using multiple sequence alignment methods by using BioEdit software. The Molecular Evolutionary Genetics Analysis version 7 (MEGA 7) was utilized to construct the phylogenetic by neighbor‐joining (NJ) method (Law et al. 2018). The identified strain was designated as *Streptomyces* sp. strain WSN‐2, and its 16S rRNA sequence was submitted to NCBI GenBank and accession no. (MN128377.1) was obtained.

### 2.3 Biosynthesis of Nanoparticles

**Figure 1:**
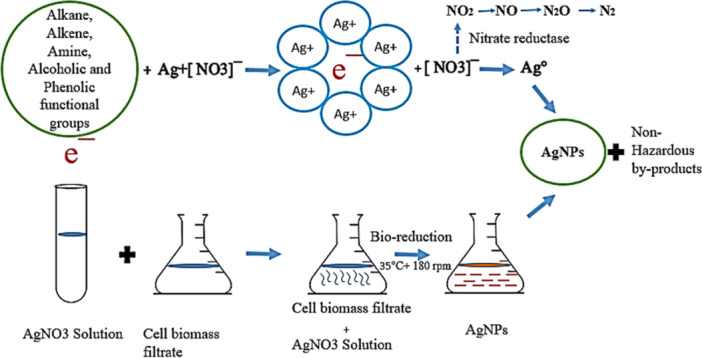
Schematic representation of AgNPs synthesis by biomass filtrate of *Streptomyces* sp. WSN‐2.

*Streptomyces* sp. strain WSN‐2 was inoculated in a 250 ml Erlenmeyer flask containing enrichment media (0.05% K_2_HPO_4_, 0.3% Casein, 1% Starch, 1% Malt extract, 0.1% Peptone, 0.1% Yeast extract, pH 7.2–7.6 in 1000 ml distilled water) and incubated for 3 days in a rotary shaker at 30°C and 200 rpm. The resulting growth was used as a seed culture for further inoculation in a 250 mL Erlenmeyer flask with starch nitrate broth (1% Starch, 0.2% KNO_3_, 0.2% K_2_HPO_4_, 0.2% NaCl, 0.03% Casein, 0.005% MgSO_4_.7H_2_O, 0.002% CaCO_3_, 0.001%FeSO_4_.7H_2_O, pH 7.2–7.6 in 1000 mL distilled water) and incubated for 7–9 days under shaking conditions at 30°C and 200 rpm (Abd‐Elnaby et al. 2016). The harvested cells were then centrifuged at 6000 × g for 15 min at 4°C. The filtrate was discarded while, cell biomass was washed thrice with distilled water to ensure the removal of media components. About 10 g of cell biomass was added to the 100 mL distilled water for 72 h at 30°C and 200 rpm. Cell biomass was separated by using filter paper and the biomass filtrate was used as a biological reducing agent. For the synthesis of AgNPs, the biomass filtrate (80 mL) was mixed with 20 mL of 3 mM silver nitrate solution and incubated at 35°C for 72 at 180 rpm in dark conditions. It is hypothesized that the presence of different bioactive functional and biochemical components is responsible for the successive reduction of Ag ions into AgNPs, such as nitrate reductase is involved in the reduction of silver ion (Ag^+)^ into neutral silver atom (Ag^°^) as shown in Figure 1.

### 2.4 Pathogenic Indicators and Antimicrobial Bioassays

The indicator bacterial test strains including *Bacillus subtilis*, *Salmonella typhi*, *Pseudomonas aeruginosa*, and *Escherichia coli* were subjected to testing the antagonistic activity of biosynthesized AgNPs. Fresh colonies of these pathogenic strains were inoculated in Tryptic Soy Broth (Oxoid Ltd., England) for overnight incubation. Following 16 h of growth at 37°C, freshly grown test bacterial cultures (25 µl) were spread on separate LB plates in triplicates. Then, wells of 3–4 mm were made by using sterile 1 mL tip and were filled with 100 µL of AgNPs. The plates were incubated at 30°C for 24 h. The appearance of clear zones indicated the antibacterial activities. The diameter of zone of growth inhibition of each test strain in triplicate were measured. Streptomycin (60 µg/mL) served as a positive control in this experiment. Negative controls using only the biomass filtrate and the silver nitrate solution were also prepared.

Additionally, the antifungal activity of synthesized AgNPs derived from the biomass filtrate of *Streptomyces* sp. WSN‐2 was assessed against different pathogenic fungal strains (*Aspergillus niger*, *Aspergillus fumigatus*, *Aspergillus flavus*, and *Fusarium oxysporum)*. The fresh suspension of these fungal strains were prepared by mixing spores taken from pure colonies into sterilized water. Potato dextrose agar (PDA) plates were prepared and was punctured evenly with a cork borer to create wells with a diameter of 3–4 mm (Valsalam et al. 2019). About 25 µL of each fungal spore suspension was spread onto PDA plates and holes were filled up with 100 µL of AgNPs colloid solution (at a concentration of 60 µg/ml). The plates were then, incubated at 30°C for 48–72 h. For comparison purposes, an antifungal agent (amphotericin B, 5 mg) was diluted in sterilized deionized water to achieve the same concentration (60 µg/mL) as the AgNPs and used as positive control for antifungal activity. The plates were kept at 30°C ± 2°C for 48–72 h until the appearance of zone of inhibitions. The diameter of zone of growth inhibition in triplicate were measured using a scale, and the respective values for the AgNPs, negative and positive controls for antibacterial and antifungal assays were also documented. All the antimicrobial assays were conducted in triplicate. Microsoft Excel XP 2010 was used to calculate the readings as the mean of standard deviation (±) in three replicates.

### 2.5 AgNPs Analysis

#### 2.5.1 UV‐Vis Spectroscopy Studies

The nanoparticles were initially confirmed using UV‐Vis spectroscopy analysis conducted with a spectrophotometer (NanoDrop 2000, Thermo Scientific, USA) within the wavelength range of 300 nm to 700 nm. This method was employed to determine the maximum value of SPR (Abirami and Kannabiran 2016).

#### 2.5.2 Fourier Transform Infrared (FT‐IR) Spectroscopy Studies

FT‐IR analysis was conducted to ascertain the presence of active functional groups on the surface of AgNps, which play a role in the reduction, stabilization, and capping of these particles. The FT‐IR spectra were acquired using the IR‐tracer 100 (SHIMADZU) model spanning a range from 4000 to 500 cm^−1^ (Al‐Dhabi et al. 2019).

#### 2.5.3 TEM Studies

The dimensions and morphology of biosynthesized AgNPs were characterized using TEM (JEM‐1230 model by JEOL, Japan), operated at an acceleration voltage of 120 kV. For the spectroscopic investigation, a droplet of AgNPs colloids was applied onto a carbon‐coated copper grid (Sanjivkumar et al. 2019).

#### 2.5.4 SEM Studies

SEM was employed to examine the shape, morphology, and dimensions of the resulting nanostructures. A specimen for SEM analysis was prepared by depositing a droplet of the suspension onto carbon‐coated copper grids. Excess solution was carefully removed, and the grids were left to air dry at room temperature. The SEM imaging was performed using a JSM‐6480 scanning electron microscope manufactured by JEOL, Japan, operating at an accelerating voltage of 20 keV (Sivasankar et al. 2018).

#### 2.5.5 Energy Dispersive X‐Ray (EDX) Spectroscopic Analysis

EDX was conducted to perform elemental analysis. For this purpose, a scanning electron microscope JSM‐6480 (JEOL, Japan) was equipped with an EDAX detector, utilizing an accelerating voltage of 20 keV (Al‐Dhabi et al. 2019).

#### 2.5.6 Determination of Zeta Potential

The charge stability of AgNPs was assessed by determining the zeta potential value of the colloidal solution. To measure the zeta potential, the AgNPs solution was mixed with distilled water and subjected to sonication for 10 min to disperse the particles effectively. The zeta potential was then measured using a Zeta sizer instrument (Malvern, UK). Specifically, 2.5 mL of the colloid solution was placed in the cuvette, with applied parameters of viscosity at 0.7 cp, refractive index at 1.2, and temperature maintained at 25°C. The average value of five zeta runs was calculated for subsequent analysis.

### 2.6 Determination of MIC

The MIC of AgNPs was determined using the standard micro‐dilution method according to the Clinical and Laboratory Standards Institute (CLSI) guidelines (Miller et al. 2005). A stock solution of 0.5 mg/mL AgNPs was prepared and serially diluted to obtain different concentrations between 7 and 100 µg/mL. For the experimental procedure, 0.5 ml of each dilution was mixed with 2 mL of nutrient broth in a test tube, followed by the addition of 0.5 mL of an old bacterial culture. The test tubes were then incubated at 30°C for 24 h and bacterial growth was assessed by measuring the optical density at 600 nm using a UV‐Vis spectrophotometer (NanoDrop 2000, Thermo Scientific, USA). The test tube that showed the least turbidity indicated the minimum MIC value of AgNPs. A similar procedure was used to determine the MIC against fungi, with the nutrient broth alone serving as a control.

### 2.7 H_2_O_2_ Scavenging Activity

One of the most important applications of AgNPs was their H_2_O_2_ scavenging potential, which was determined by an experimental procedure with slight modifications (Bhakya et al. 2016). A 40 mM H_2_O_2_ solution was prepared by combining H_2_O_2_ solvent with freshly prepared phosphate buffer (0.1 M, pH 7.5). Different concentrations of AgNPs (10, 20, 30, 40, 50 µg/mL) were added to 0.60 ml of the H_2_O_2_ solution and incubated at 20°C for 20 min. The absorbance after incubation was then measured using a spectrophotometer at 230 nm. Ascorbic acid was used as a standard for comparative analysis, while phosphate buffer without H_2_O_2_ served as a blank. The scavenging activity of H_2_O_2_ was calculated using the following formula (Gülçin et al. 2004):

$$\%\text{Scavenging}\left(H_{2}O_{2}\right)=\left(A_{o}-A_{1}\right)/A_{o}\times 100,$$

where *A*_o_ is the absorbance of the control and *A*_1_ is the absorbance of the sample.

## 3 Results

### 3.1 Screening and Identification of Streptomyces sp

**Figure 2:**
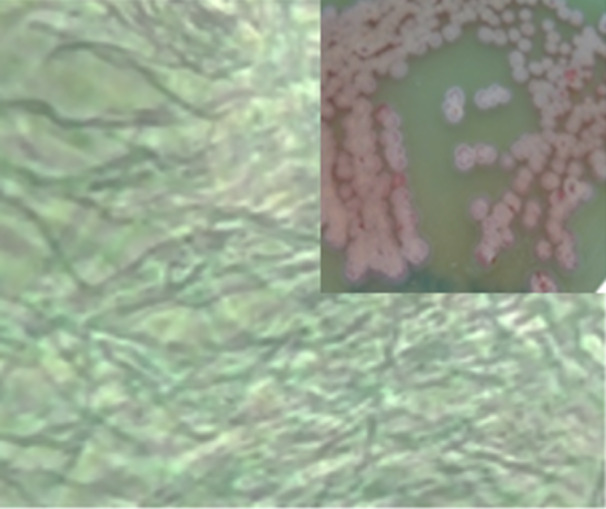
Colony morphology and phase contrast image of the *Streptomyces* sp. strain WSN‐2.

Based on the morphological characterization, the strain can be classified as an aerobic Gram‐positive actinomycete. It exhibited robust growth on the YCA medium. The aerial mycelium displayed a reddish‐pink hue, while the mycelium on agar medium produced a yellowish‐brown pigment (Figure 2).

**Figure 3:**
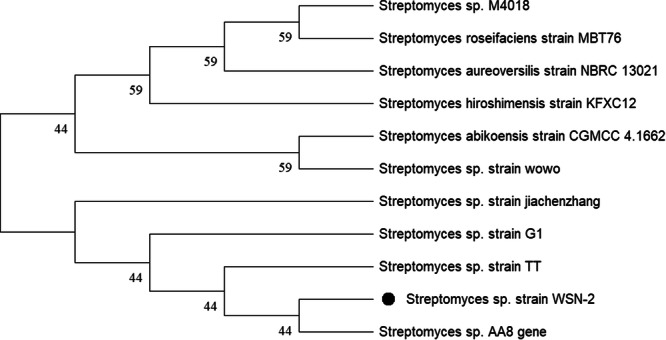
Phylogenetic comparison of *Streptomyces* sp. strain WSN‐2 based on 16S rRNA sequence by neighbor‐joining (NJ) method. The tree was constructed using the NJ method and numbers at each node represent the levels (%) of bootstrap support from 500 resampled datasets. Only value above 40% are shown in phylogenetic tree. The tree was contracted by MEGA X.

In addition, rRNA gene sequence analysis of the strain *Streptomyces* sp. WSN‐2 was performed, which showed 100% sequence similarity with known *Streptomyces* species. The nucleotide base pairs were then submitted to NCBI GenBank under the accession number MN 128377. Figure 3 shows the phylogenetic comparison of *Streptomyces* sp. strain WSN‐2 with previously identified *Streptomyces* sp. strain AA8.

### 3.2 Biological Synthesis of AgNPs

**Figure 4:**
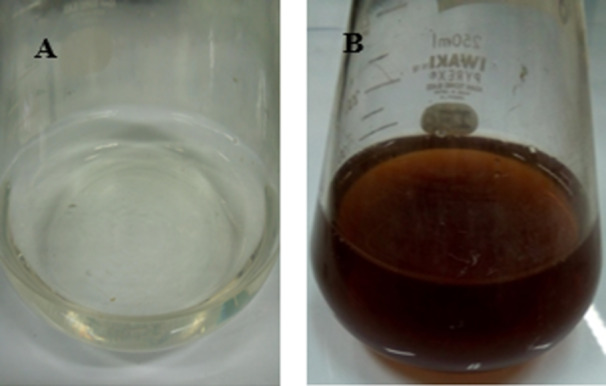
Biosynthesis of silver nanoparticles by *Streptomyces* sp. strain WSN‐2 in shake flsk. A: Biomass filtrate as control; B: Color change to dark brown indicates the synthesis of AgNPs.

AgNPs were biosynthesized by a Streptomyces‐mediated extracellular method using the cell biomass filtrate of Streptomyces sp. strain WSN‐2. Consistent with previous studies (Lachmapure et al. 2017; Vijayabharathi et al. 2018; Sheik et al. 2019) the formation of AgNPs was evidenced by the appearance of brown color in the AgNO3‐treated flasks, a deviation from the original colorless solution (Figure 4). In contrast, the control flasks, which contained only biomass filtrate, exhibited no discernible change in color. This color change is likely due to the reduction of Ag ions facilitated by the presence of reducing secondary metabolites in the biomass filtrate of Streptomyces sp. strain WSN‐2. These reducing agents not only facilitate the reduction process but also serve as stabilizers that prevent the resulting particles from agglomerating. This stabilization ensures that the bioactive potential is maintained over a longer period.

### 3.3 UV‐Visible Spectroscopy

**Figure 5:**
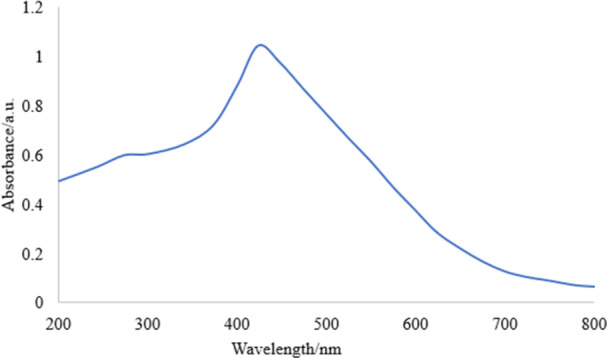
UV‐Vis. Spectroscopy analysis of AgNPs synthesized by *Streptomyces* sp. strain WSN‐2.

The changes in the optical properties of NPs are closely related to their shape and size, which are reflected in specific absorption values. A clear indication of the synthesis of AgNPs is the transformation of a colorless solution into a brown hue. The free electrons in the AgNPs absorb easily visible light and change to a higher energy state. However, this state is unstable, which causes them to revert to their base energy level and emit photons (Shafiqa et al. 2018). The marked changes in SPR are directly influenced by the shape, size, and environmental conditions in which the NPs are located. *Streptomyces* sp. strain WPS‐2 exhibited a maximum SPR at 423 nm, a characteristic peak for AgNPs (Figure 5).

### 3.4 FTIR

**Figure 6:**
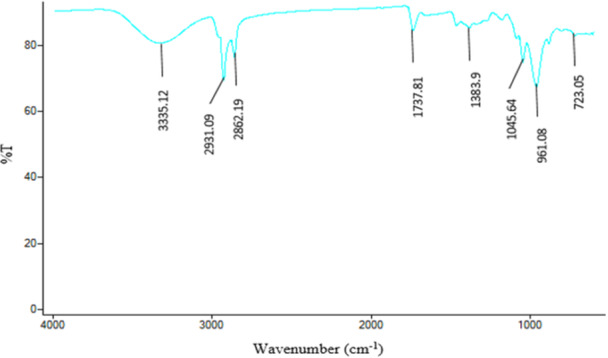
FTIR analysis of AgNPs synthesized by *Streptomyces* sp. strain WSN‐2.

The identification of various functional groups that play a role in the transformation of inorganic salts into a specific elemental component through processes such as reduction, stabilization, and capping was determined by FTIR analysis (Figure 6). This infrared spectroscopic analysis is directly related to the detection of vibrational and rotational motions of these functional groups. In particular, the peak at 732 cm^−^^1^ corresponds to aromatic C‐H bending vibrations, while the peaks at 961 and 1046 cm^−1^ indicate the presence of C‐N and C‐O groups, respectively. In addition, the peaks at 1384 and 1738 cm^−1^ confirm the stretching of C‐H (alkane bending) and C = C bonds, respectively. Peaks in the range of 2800 to 3000 cm^−1^ indicate C‐H (alkane stretch), and the broad peak at 3335 cm^−1^ is attributed to the stretch vibrations of the O‐H groups of alcohol/phenol and the N‐H groups (primary amine stretch) (Abd‐Elnaby et al. 2016; Vijayabharathi et al. 2018).

### 3.5 SEM

**Figure 7:**
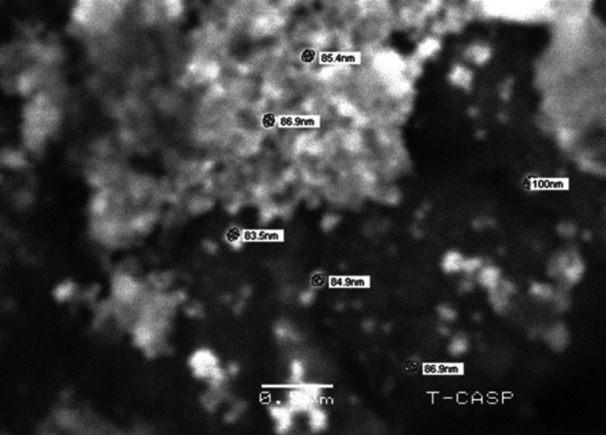
SEM analysis of AgNPs synthesized by *Streptomyces* sp. strain WSN‐2.

SEM analysis was performed to evaluate the size and morphology of the biosynthesized AgNPs. The AgNPs with size range from 0.83 to 100 nm with a smooth surface texture and a spherical shape were observed (Figure 7). Furthermore, they were also uniformly distributed and showed consistent growth patterns, as shown in Figure 7.

### 3.6 Energy‐Dispersive X‐Ray Analysis (EDX)

**Figure 8:**
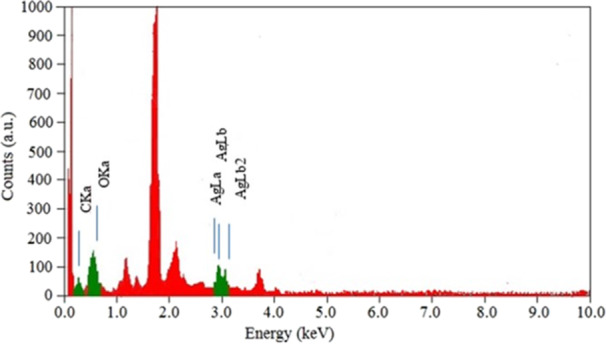
EDX spectrum of AgNPs synthesized by *Streptomyces* sp. strain WSN‐2.

EDX analysis is a valuable analytical method to evaluate the elemental composition and chemical properties of samples. In this study, EDX was used to verify the presence of elemental silver in the sample. In particular, the optical absorption peaks indicated the SPR of Ag metal at energy values between 3 and 4 keV. These distinct peaks indicate X‐ray absorption by metallic silver nanocrystallites. Other observed peaks corresponding to carbon and oxygen indicate the presence of precipitates or residual materials surrounding the nanoparticles (Figure 8).

### 3.7 TEM

**Figure 9:**
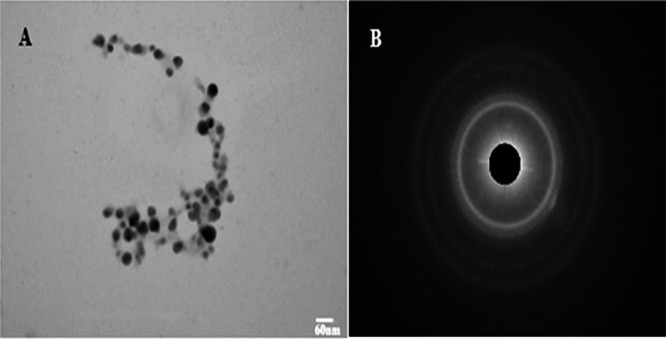
Confirmation of silver nanoparticles (AgNPs) synthesis by TEM and SAED. A: TEM analysis of synthesized AgNPs from *Streptomyces* sp. strain WSN‐2; B: SAED pattern for the crystalline confirmation of AgNPs.

The synthesized AgNPs were analyzed by TEM and showed spherical particles that were well dispersed and had a diameter of 50–60 nm (Figure 9a). Similar studies on TEM characterization of AgNPs have also shown a spherical morphology in the range of 10–60 nm (Abd‐Elnaby et al. 2016; Goudarzi et al. 2016). The selected area electron diffraction (SAED) pattern further confirms the crystallinity and spherical shape of AgNPs, as evidenced by the formation of distinct bright rings (Figure 9b).

### 3.8 Zeta Potential Analysis

**Figure 10:**
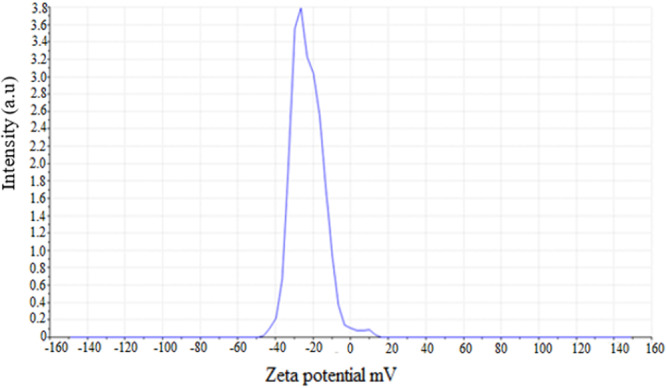
Zeta potential of of AgNPs synthesized by *Streptomyces* sp. strain WSN‐2.

The surface charge stability of the biologically synthesized AgNPs was evaluated by determining the zeta potential value of the particles. The measured zeta potential of the biosynthesized AgNPs (−22.9 mV) indicates a net negative surface charge and a moderate electrostatic repulsion between particles, which supports colloidal stability under the synthesis/storage conditions used (Figure 10).

### 3.9 Antibacterial Activity

**Figure 11:**
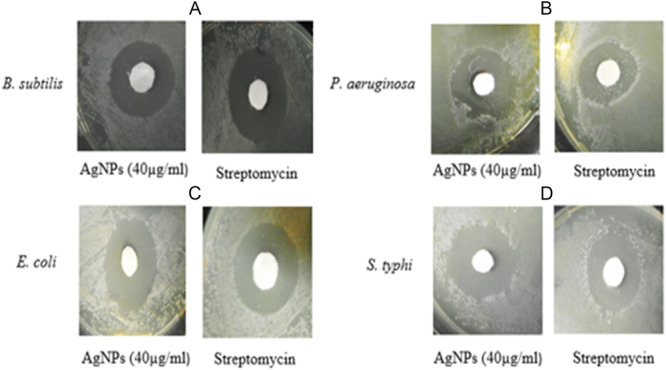
Antibacterial activity of AgNPs synthesized by *Streptomyces* sp. strain WSN‐2 against (A) *B. subtilis*, (B) *P. aeruginosa*, (C) *E. coli*, and (D) *S. typhi* in the comparison to positive streptomycin.

The antibacterial potential of the synthesized AgNPs at a concentration of 40 µg/ml was investigated and the results are summarized in Table 1. The results indicate remarkable antibacterial efficacy, with maximum inhibition observed against gram‐positive *Bacillus subtilis* (21 ± 1.53) and gram‐negative *Salmonella typhi* (23 ± 1.0). Significant antibacterial activity was also observed against *Pseudomonas aeruginosa* (19 ± 1.15) and *Escherichia coli* (20 ± 1.15) at the same concentration. Streptomycin was used as a standard reference, and at a concentration of 40 µg/ml, the diluted antibiotic exhibited slightly better antibacterial potential than the AgNPs. The diameters of the inhibition zones for *P. aeruginosa, E. coli, B. subtilis*, and *S. typhi* were recorded as (22 ± 1.53, 23 ± 1.25, 23 ± 1.73, 24 ± 1.53), respectively (Figure 11). These results strongly suggest that the AgNPs synthesized from the biomass filtrate of *Streptomyces* sp. strain WSN‐2 have promising antimicrobial activity against a wide range of microbial pathogens.

**Table 1:** Antibacterial activity of synthesized AgNPs from *Streptomyces* sp. WSN‐2 as compared to the positive control streptomycin.

Test pathogens	AgNPs (4 µg/mL) Inhibition zone(mm)	Streptomycin (40 µg/mL) Inhibition zone(mm)
*P. aeruginosa*	19 ± 1.15^ab^	22 ± 1.53 ^cd^
*E. coli*	20 ± 1.15^ab^	23 ± 1.25^cde^
*B. subtilis*	21 ± 1.53^b^	23 ± 1.73^cde^
*S. typhi*	23 ± 1.00^cde^	24 ± 1.53^e^

*Note:* The experiments were conducted in triplicate, and Microsoft Excel XP 2010 was used to calculate the readings as the mean of standard deviation (±) in three replicates. One way ANOVA (*p* < 0.05 was used to calculate the significant differences between the values). Different letters indicate significantly different the values.

### 3.10 Antifungal Activity

**Figure 12:**
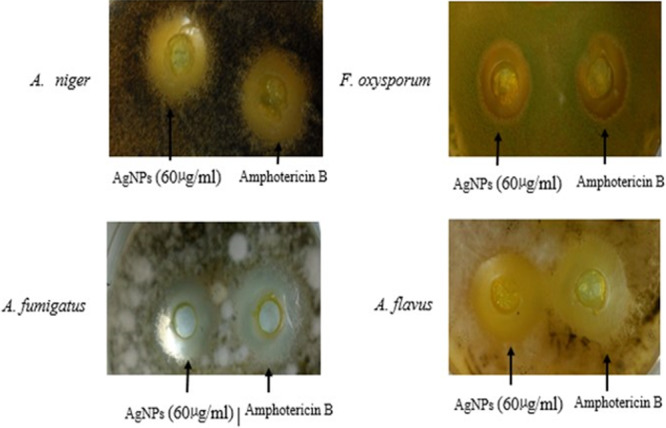
Antifungal activity of of AgNPs synthesized by *Streptomyces* sp. strain WSN‐2 against various fungal pathogens in comparison to amphotericin as positive control.

The AgNPs synthesized from *Streptomyces* sp. strain WSN‐2 were evaluated for their antifungal activity against various fungi (*A. niger*, *A. flavus*, *F. oxysporum* and *A. fumigatus*) using the well diffusion assay method, with amphotericin serving as a positive control. The results showed that the synthesized nanoparticles exhibited remarkable antifungal efficacy, with the highest activity observed against *A. flavus* (16 ± 1.15). They also showed significant activity against *F. oxysporum*, *A. fumigatus* and *A. niger*, with inhibition zone diameters of 14 ± 1.53, 15 ± 1.70, and 16 ± 1.25, respectively (Table 2). Of particular importance in this assay was the observation that AgNPs at a concentration of 60 µg/mL showed considerable antifungal activity against the major plant pathogenic fungi, comparable to the standard antifungal agent amphotericin B. Amphotericin B exhibited maximum activity against *A. flavus* (17 ± 1.25) and *A. fumigatus* (17 ± 1.25), while it also showed significant activity against *F. oxysporum* (16 ± 1.53) and *A. niger* (18 ± 1.41) (Figure 12). So, this study highlights the potential of AgNPs synthesized from *Streptomyces* sp. strain WSN‐2 as effective antifungal agents against a range of pathogenic fungi.

**Table 2:** Antifungal activity of bio‐synthesized AgNPs from biomass filtrate of *Streptomyces* sp. strain WSN‐2 with the comparison of standard positive control amphotericin.

Test pathogen	AgNPs (60 µg/mL) inhibition zone (mm)	Amphotericin B (60 µg/mL) inhibition zone (mm)
*F. oxysporum*	14 ± 1.53^a^	16 ± 1.53^b^
*A. fumigatus*	15 ± 1.70^ab^	17 ± 1.25^c^
*A. niger*	16 ± 1.25^bc^	18 ± 1.41 ^cd^
*A. flavus*	16 ± 1.15^bc^	17 ± 1.25 ^cd^

*Note:* The experiments were conducted in triplicate, and Microsoft Excel XP 2010 was used to calculate the readings as the mean of standard deviation (±) in three replicates. One way ANOVA (*p* < 0.05 was used to calculate the significant differences between the MICs). Different letters indicate significant difference between the values.

### 3.11 The MIC of AgNPs

The biosynthesized AgNPs demonstrated notable antibacterial efficacy, particularly against *Pseudomonas aeruginosa* with a MIC (Minimum Inhibitory Concentration) of 8.00 µg/mL. Conversely, the MIC against *Aspergillus niger* was recorded at 14.50 µg/mL. The observed MIC values against *Escherichia coli* (10.50 µg/mL), *Salmonella typhi* (11.00 µg/mL), and *Bacillus subtilis* (13.00 µg/mL) suggest comparatively lower activity against gram‐positive strains. Additionally, the nanoparticles exhibited promising activity against other pathogenic fungal strains, including *Aspergillus flavus* (15.00 µg/mL), *Fusarium oxysporum* (16.50 µg/mL), and *Aspergillus fumigatus* (18.00 µg/mL), as detailed in Table 3.

**Table 3:** MIC of biosynthesized AgNo3 nanoparticles against different bacterial and fungal pathogens.

Test pathogens	MIC values (µg/mL)
*P. aeruginosa*	8.00 ± 0.05^ab^
*E. coli*	10.50 ± 0.06^ab^
*S. typhi*	11.00 ± 0.09^bc^
*B. subtilis*	13.00 ± 0.01 ^d^
*A. niger*	14.50 ± 0.05^de^
*A. flavus*	15.00 ± 0.05^ef^
*F. oxysporum*	16.50 ± 0.07^fg^
*A. fumigatus*	18.00 ± 0.07 ^h^

*Note:* The experiments were conducted in triplicate, and Microsoft Excel XP 2010 was used to calculate the readings as the mean of standard deviation (±) in three replicates. One way ANOVA (*p* < 0.05 was used to calculate the significant differences between the MICs values).

### 3.12 H_2_O_2_ Scavenging Activity

**Figure 13:**
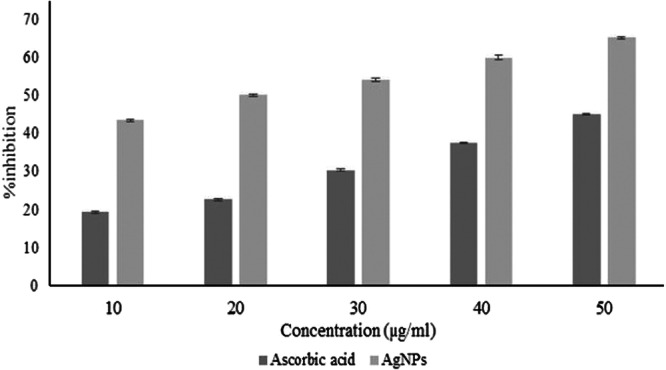
H_2_O_2_ scavenging effect of of AgNPs synthesized by *Streptomyces* sp. strain WSN‐2 in comparison to ascorbic acid.

The H_2_O_2_ scavenging activity of AgNPs was evaluated with different concentrations of NPs (10, 20, 30, 40, and 50 µg/mL), using l‐ascorbic acid as a standard reference. At a concentration of 50 µg/mL, the AgNPs showed a significant activity of approximately 65.2%, exceeding the scavenging potential of standard L‐ascorbic acid of 45.1% (Figure 13). This showed that increasing the concentration of AgNPs leads to an increase in scavenging activity up to a certain threshold. In addition, the AgNPs were involved in the formation of ROS in the presence of H_2_O_2_, which accelerated the dissolution of AgNPs even at lower concentrations.

## 4 Discussion

Nanoparticles, particularly AgNPs, have taken attention for their unique properties and broad applications in fields such as environmental, agricultural, and medical. Traditional physical and chemical synthesis methods faced limitations including high costs, energy consumption, and toxic byproducts, making them unsuitable for biological systems. The present study successfully demonstrated biosynthesis of AgNPs using a novel *Streptomyces* sp. strain WSN‐2. The identification of this strain, confirmed by 100% sequence similarity of 16S rRNA gene with known *Streptomyces* species using nucleotide Blastn analysis and the sequence was deposited in GenBank under accession MN128377. Prior studies have been emphasizing the diverse biosynthetic capabilities of *Streptomyces* spp (Shaikh et al. 2021; Nguyen et al. 2023). for the synthesis of nanoparticles. The extracellular biosynthesis approach yielded a distinct brown coloration upon AgNO₃ treatment, confirming nanoparticle formation, in agreement with previous findings (Strużyńska and Skalska 2018; Ahmed et al. 2020). UV‐Vis spectroscopic analysis revealed a characteristic SPR peak at 423 nm, comparable to prior reports where *Streptomyces*‐derived AgNPs showed peaks between 420 and 430 nm (Jain et al. 2021; Mohanta and Behera 2014), confirming nanoparticle stability and formation.

FTIR analysis identified functional groups such as O–H, N–H, C = O, and aromatic C–H, suggesting the role of proteins, phenolics, and amines in reducing and capping AgNPs. These results corroborate with previous FTIR‐based studies that proposed similar functional groups as capping agents (Singh and Mijakovic 2022). The presence of spherical, well‐dispersed particles in the size range of 50–60 nm, as observed via SEM and TEM, falls within the typical range reported for biosynthesized AgNPs (Somda et al. 2024), while SAED patterns confirmed their crystallinity.

The EDX analysis confirmed elemental silver with SPR‐related peaks between 3 and 4 keV, aligning with existing studies (Sheik et al. 2019), while additional peaks indicated residual capping agents. The observed zeta potential of −22.9 mV indicates moderate nanoparticle stability, comparable to values reported by others for biosynthesized AgNPs stabilized by microbial metabolites (Vijayabharathi et al. 2018). Many authors reported that biologically synthesized nanoparticles exhibited negative zeta potentials because proteins, polysaccharides and phenolics from extracts or microbial secretions cap the AgNPs and expose negatively charged functional groups at the particle surface. This capping both reduces aggregation and contributes to the measured negative zeta (Sati et al. 2025; Azeeze et al. 2021). It has been reported that zeta magnitude of ±30 mV or greater is usually considered the threshold for highly stable nano‐suspensions. Therefore −22.9 mV should be read as “moderately stable” and likely sufficient for short‐to‐medium term storage (Serrano‐Lotina et al. 2023). These observations confirms the effectiveness of *Streptomyces* sp. WSN‐2 biomass filtrate as a reducing and stabilizing agent for the nanoparticles, thereby facilitating the stabilization of the charges on the surfaces of the nanoparticles.

The AgNPs showed broad‐spectrum antimicrobial efficacy, significantly inhibiting both Gram‐negative and Gram‐positive bacteria. Notably, *Salmonella typhi* and *Bacillus subtilis* showed inhibition zones of 23 ± 1.53 and 21 ± 1.00 mm, respectively, which were only slightly lower than those of streptomycin (Table 1). These results supports the findings of Singh et al (Singh et al. 2015)., where *Streptomyces*‐derived AgNPs showed higher efficacy against Gram‐negative strains due to their thinner peptidoglycan layer facilitating nanoparticle penetration.

The antifungal efficacy observed in the present study (Table 2) is noteworthy, with *A. flavus* and *A. niger* showing inhibition zones up to 16 ± 1.15 mm, which are quite comparable to amphotericin B (17–18 mm). These results surpass those of several earlier biosynthetic studies, where inhibition zones for fungal strains often remained under 12 mm (Abd‐Elnaby et al. 2016), suggesting the high bioactivity of the AgNPs biosynthesized by *Streptomyces s*p. WSN‐2.

MICs further validated the potency of these AgNPs, particularly against *P. aeruginosa* (8 µg/mL), which exhibited the lowest MIC, suggesting high susceptibility (Table 3). This is consistent with prior reports indicating that *P. aeruginosa* is particularly vulnerable to nanoparticle‐induced oxidative stress and membrane disruption (Lee et al. 2019).

The biosynthesized AgNPs also displayed potent H₂O₂ scavenging activity, peaking at 65.2% at 50 µg/mL surpassing the standard antioxidant l‐ascorbic acid (45.1%). This indicates the nanoparticles' potential as ROS scavengers, a feature also reported by Ahmed et al (Ahmed et al. 2020). who observed strong antioxidant properties of microbial AgNPs due to the presence of polyphenolic capping agents. So, the current findings highlight the potential of *Streptomyces* sp. strain WSN‐2 as a valuable biogenic source for the green synthesis of AgNPs with promising antibacterial, antifungal, and antioxidant properties as compared to chemically synthesized AgNPs, However, the study's findings are constrained by the lack of cytotoxicity evaluation on mammalian cells, and by the absence of any *in vivo* validation. Although green‑synthesized AgNPs are often regarded as more biocompatible, reviews and experimental reports have repeatedly documented significant cytotoxicity of AgNPs, depending on size, dose, exposure duration, and surface chemistry. Without efficient cytotoxicity assays, claims of “non‑toxic” or “safe” AgNPs remain hypothetical (Amar kumar et al. 2024; Li et al. 2022). Moreover, antimicrobial activity observed *in vitro* does not necessarily predict safety or efficacy in living organisms, where bio‐distribution, clearance, immune responses, and organ accumulation may alter both benefit and risk.

Likewise, generation of ROS or silver‑ion release to explain microbial killing and antioxidant activity also remain hypothetical without direct mechanistic evidence. Prior studies often link cytotoxicity of AgNPs to oxidative stress, membrane disruption, or DNA damage but such effects vary by nanoparticle size, shape, and capping agents (Ahmad et al. 2024). To support any mechanistic hypothesis, future experiments should include ROS measurements, assays for Ag⁺ release under the experimental conditions, and tests of whether, ROS scavengers or metal‑ion chelators reduce antimicrobial or antioxidant effects. Additionally, regarding membrane integrity or electron microscopy studies of treated microbes/cells would be helpful to clarify how these AgNPs interact with biological targets.

## 5 Conclusion

The current study conducted on the synthesis of AgNPs using the biomass filtrate of *Streptomyces* sp. strain WSN‐2, highlights several key findings. Firstly, the process is deemed eco‐friendly, cost‐effective, and environmentally safe, showcasing its potential as a sustainable method for AgNP synthesis. Furthermore, the evaluation for the antibacterial and antifungal properties of these biogenic AgNPs indicated their effectiveness against several microbial pathogens. The observed significant H_2_O_2_ scavenging activity is notable as it contributes to maintaining cellular balance by regulating ROS, thereby supporting proper cellular function and organelle health. The promising attributes of biogenic AgNPs synthesized by *Streptomyces* sp. WSN‐2, is their antimicrobial properties against diverse range of bacterial and fungal pathogens, making them as valuable candidates for future applications as antimicrobial agents. However, *in. vivo* studies are required to for validation of the results. To support any mechanistic hypothesis, future experiments should include ROS measurements, assays for Ag⁺ release under the experimental conditions, and tests of whether, ROS scavengers or metal‑ion chelators reduce antimicrobial or antioxidant effects are required.

## Author Contributions

**Muhammad Sultan Anjum:** performed the experiments, analyzed the data, writing – original draft of the manuscript. **Shazia Khaliq:** supervision, methodology development, data analysis, manuscript – review and editing. **Neelma Ashraf:** validation; writing – review and editing; formal analysis; visualization. **Munir Ahmad Anwar:** data interpretation, manuscript revision. **Kalsoom Akhtar:** data visualization and validation.

## Funding

The authors received no specific funding for this work.

## Ethics Statement

The authors have nothing to report.

## Conflicts of Interest

None declared.

## Data Availability

The data that support the findings of this study are available on request from the corresponding author. The data are not publicly available due to privacy or ethical restrictions.
